# A Piece of Bone and Teeth, Which Were Dissected from between Two Layers of Membrane Forming the Walls of an Ovarian Cyst, Taken from a Girl after Death

**Published:** 1851-01

**Authors:** 


### ARTICLE XVI.

### *A Piece of Bone and Teeth, which were dissected from between two Layers of Membrane forming the Walls of an Ovarian Cyst, taken from a Girl after death.*

Eliza F——, aged twenty-two, single, was admitted at the Stoke Newington Dispensary, under Dr. Duesbury, Aug. 9th, 1850, with dropsical swellings, distending so uniformly the abdominal parietes, that it was difficult to diagnose whether it was ovarian disease or ascites. She complained of weakness and inconvenience from the weight and size of the body. She stated that she had been ailing for three years and upwards, during which period she had suffered much from pain in the left side of the abdomen, but had not perceived her body enlarging more than two years; that it had increased much more rapidly of late. She attended at the dispensary, and slightly improved in health, under tonics and diuretics, until a fortnight previous to her death, when she was attacked with peritonitis, and died on the 7th October. Mr. Price attended her for a considerable period for what he considered to be an ovarian tumor; and four months previous to her being admitted a patient at the dispensary, (in consequence of the lady with whom she lived as servant having suspicions of her being pregnant,) he sent her to Dr. Lee, who pronounced the uterus to be unimpregnated, and confirmed the previous diagnosis as to the existence of an ovarian tumor. On opening the abdomen, the left ovarium was found to have been converted into a cyst, occupying the greater portion of that cavity, the viscera of which were pressing in every direction. A few recently deposited threads of lymph extended from its anterior surface to the peritoneum, lining the abdominal parietes ; otherwise it was perfectly detached, except by its peduncle, which was not larger than the little finger, and consisted merely of the obliterated Fallopian tube, enlarged vessels and peritoneum. The cyst, together with the uterus and its appendages, were removed; and on opening the cyst, from three to four gallons of a cream-colored opaque fluid escaped, suspended in which were numerous flakes of fatty matter; it also contained a conglomerate mass as large as the fetal head at six months, consisting of hair (from four to five inches long) and this fatty substance matted together, besides many smaller pieces of the same substance. The cyst was divided by transverse bands, and also into pouches or smaller cysts, opening into the common cavity; adhering to the walls in places, were considerable quantities of the fatty matter, and attached hair, similar to that in the mass; and between two layers of the membrane forming the walls of the cyst, on the left side, was the specimen produced. The teeth are in every respect perfect, with their fangs inserted into the processes of the bone, resembling the alveolar processes of the jaws. The right ovarium was rather larger than usual, congested, and contained several small cysts, the size of a pea, filled with fatty matter. The uterus presented the appearance, in all respects, of that of a virgin, and healthy, with the exception of slight congestion, which might be merely post-mortem.

Dr. Bailie mentions similar fatty matter, hair, bone, and the rudiments of teeth, (but without fangs,) having been found in ovarian tumors, and under circumstances leaving little doubt that they had formed independently of impregnation. One case occurred in his own practice. In another, published in the *Philosophical Transactions*, this change was found in the ovarium of a child, whose age did not exceed twelve or thirteen years, with the hymen perfect, the uterus not increased in bulk, as is usual at puberty, together with the other signs of puberty wanting. From the fact of the uterus being totally devoid of all those changes that follow impregnation, whether the fetus be developed within its cavity, or, as in extra-uterine fetation, within the ovarian or Fallopian tube, from the respectability of the girl's parents, together with the good character she herself had borne, and her anxious desire that her body should be opened, for the satisfaction of her friends, in consequence of the suspicion her appearance had excited, Mr. Denny was induced to believe that in this case there had been no impregnation. The consideration of this case suggests whether an ovum which the stimulus of the catamenial period caused to burst from the Graafian vesicles, and which was prevented from passing off with the menstrual discharge by the impermeability of the Fallopian tube, might not become adherent to the lining membrane of the ovary, or Fallopian tube, and thus obtain nourishment, setting in action a power which may be inherent in it, of developing the structures of the body, but in an incoherent manner, without the power to regulate the formation, or to produce a circulating system, or nervous center, necessary to the perfect development and independent life of the fetus; the latter power may emanate from the male, exerting an influence in some way analogous to the electric influence exerted in crystallization, of attracting and arranging the particles, so as to give a definite form, and thus to characterize the individuality of a salt. The statistical information furnished by the record of these cases, especially with evidence of existing virginity, may, by affording data for physiologists, enable them, at some future period, to throw light upon the functions of the ovary, and upon the elements supplied towards the formation, and the power exercised over the development of the fetus, by the sexes individually, and may also prove of practical importance, in removing the odium which a supposition of the necessity of impregnation in such cases as the above, might cast upon the fair fame of the patient.—*London Lancet.*

